# Reviewer List

**DOI:** 10.4269/ajtmh.96-02r

**Published:** 2017-02-08

The journal would like to recognize the following individuals who have reviewed manuscripts during the past year. We thank you for your continued support.

Aagaard-Hansen, Jens

Aaskov, John

Abdel-Messih, Ibrahim

Abdelwahab, Sayed

Abeles, Shira

Abudho, Bernard

Ackerman, Hans

Adams, Lisa

Adams, Nekhonti

Adetifa, Ifedayo

Adjei, Jones

Agampodi, Suneth

Agosti, Jan

Akaike, Takaaki

Akanda, Ali

Akinwale, Olaoluwa

Alabi, Abraham

Alali, Walid

Alba-Soto, Catalina

Alcid, David

Aldstadt, Jared

Alexander, Neal

Ali, Mohammad

Aliota, Matthew

Allen, Jonathan

Allison, Jill

Almeida, Carlos

Alpern, Jonathan

Amaro, Deirdre

Amato, Valdir

Amman, Brian

Anders, Katherine

Anderson, Kathryn

Andrade, Rosa

Andre, Marcos

Andres, Sönke

Angheben, Andrea

Angus, Brian

Anisimov, Andrey

Antinori, Spinello

Antonelli, Lis

Antonini, Pietro

Appleton, Christopher

Arai, Satoru

Araújo, Stanley

Arce-Fonseca, Minerva

Arguin, Paul

Arlian, Larry

Armah, George

Armstrong, Philip

Armstrong, Derek

Armstrong Schellenberg, Joanna

Arndt, Michael

Arnold, Benjamin

Aronson, Naomi

Aronson, Judith

Aseffa, Abraham

Ashorn, Per

Ashton, Ruth

Asiedu, Kingsley

Asogun, Danny

Ateba-Ngoa, Ulysse

Auburn, Sarah

Auer, Herbert

Aunger, Robert

Avila, Luciana

Avsic-Zupanc, Tatjana

Awandare, Gordon

Aweeka, Fran

Ayaz, Muhammad

Azad, Abdu

Azman, Andrew

Babu, Subash

Bacellar, Olivia

Bacon, David

Bahr, Nathan

Bai, Ying

Bain, Rob

Baird, J. Kevin

Baird, Robert

Bairy, Indira

Bajillan, Hendren

Ball, Jacob

Baltzell, Kimberly

Bancone, Germana

Bandsma, Robert

Barbour, Alan

Barker, Christopher

Barnett, Elizabeth

Barnett, Elizabeth D.

Barrett, Alan

Bart, Jean-Mathieu

Bart, Aldert

Bartholomay, Lyric

Bassat, Quique

Bassiri-Jahromi, Shahindokht

Battle, Katherine

Bausch, Daniel

Beau de Rochars, Valery

Becker, Soeren

Beckham, David

Beier, John

Beilke, Marke

Beissner, Marcus

Bell, David

Bellomo, Carla

Belo, Silvana

Ben-Shimol, Shalom

Benard, Gil

Benavente, Luis

Benet, Thomas

Berentsen, Are

Berg, Douglas

Bergren, Nicholas

Bergstrom, Sven

Berman, Jonathan

Bern, Caryn

Bernigaud, Charlotte

Bianchin, Marino

Bibby, Kyle

Bielefeldt-Ohmann, Helle

Biggs, Holly

Billerbeck, Sonja

Biran, Adam

Biritwum, Nana Kwadwo

Black IV, William

Blacksell, Stuart

Blair, Patrick

Blanton, Ronald

Blanton, Lucas

Blitvich, Bradley

Bloch, Evan

Blum, Johannes

Bockarie, Moses

Boggild, Andrea

Bogoch, Isaac

Bogus, Joshua

Bolling, Bethany

Boni, Maciej

Borges, Aércio Sebastião

Bosilkovski, Mile

Bossart, Katharine

Boughey, Simon

Boulouis, Henri-jean

Boussaa, Samia

Boussinesq, Michel

Bouteille, Bernard

Bowen, Richard

Bowman, Dwight

Brackney, Doug

Bradley, David

Brady, Oliver

Bravo, Francisco

Brazil, Reginald

Brehm, Klaus

Breman, Joel

Brenière, Simone

Brennan, Ben

Brett-Major, David

Briese, Thomas

Brodskyn, Claudia

Brooker, Simon

Brookes, Victoria

Brooks, Yolanda

Brown, James

Brunetti, Enrico

Buccheri, Renata

Buekens, Pierre

Burza, Sakib

Bustamante, Dulce

Bustinduy, Amaya

Bustos, Javier

Caballero, Maria Rosario

Cabibbe, Andrea

Cai, Li

Calisher, Charles

Camargo, Luis Marcelo

Campbell, Karen

Cappello, Michael

Caramelo, Julio

Carmolli, Marya

Carrington, Lauren

Carroll, I.

Carter, Keith

Caruso, Bethany

Carvalho, Edgar

Casanova, Lisa

Castellanos-Gonzalez, Alejandro

Casulli, Adriano

Chaccour, Carlos

Chae, Joon-seok

Chaignat, Claire-Lise

Challenger, Joseph

Chandrashekar, Ramaswamy

Chantratita, Narisara

Chen, Yen-Chia

Chen, Zeliang

Chen, Lin

Chen, Huangchi

Chesnais, Cédric

Chieffi, Pedro

Chioccola, Vera Lucia Pereira

Choi, Young Ki

Chomel, Bruno

Chou, Deng-Wei

Chowdhury, Rajib

Christofferson, Rebecca

Chu, Helen

Chuang, Sally

Ciota, Alexander

Clapham, Hannah

Coffey, Lark

Cohen, Cheryl

Colombara, Danny

Colombo, Sandro

Conceição-Silva, Fatima

Conlon, Chris

Connor, Bradley

Conrad, Melissa

Converse, Paul

Conway, David J.

Cookson, Susan

Cooper, Roland

Corbel, Michael J

Costa, Carlos

Coulibaly, Jean

Coyle, Christine

Crane, Rosie

Croda, Julio

Croft, Simon

Crompton, Peter

Cruz, Israel

Cucunubá, Zulma

Cui, Liwang

Cunliffe, David

Cunningham, Jane

Currie, Bart

Curtis, Val

Cusick, Sarah

Cutler, Sally

da Rosa, João

da Silva, Alexandre

Dahlgren, Fredrick

Daily, Johanna

Dalrymple, Ursula

Damasceno, Lisandra

Dance, David

Dary, Omar

Dash, Aditya

Dassoni, Federica

Datiko, Daniel Gemechu

Davaalkham, Dambadarjaa

Davey, Gail

David, John

Davis, Jennifer

de Brauw, Alan

de Jong, Bouke

de Oliveira, Camila

De Rauw, Klara

de Vos, Margaretha

Debes, Amanda

Decaro, Jaston

Degarege, Abraham

Del Brutto, Oscar

del Canto, Felipe

Delea, Maryann

Deloron, Philippe

Denning, David

Depaquit, Jerome

Devine, Angela

Devine, Sue

Deye, Gregory

Dhanasekaran, Vijaykrishna

Dhorda, Mehul

Diallo, Mawlouth

Diallo, Karidia

Diaz, James

Diez, Cristina

Diniz, Pedro

Direny, Abdel

Dissanayake, Gunawardena

Dittrich, Sabine

Dobbs, Katherine

Domingo, Gonzalo

Dorn, Patricia

Dorsey, Grant

Dorta-Contreras, Alberto Juan

Drake, Tom

Drakeley, Chris

Drancourt, Michel

Drebot, Mike

Dreibelbis, Robert

Drexler, Naomi

Dubey, Vikash

Dubourg, Greg

Dubreuil, Daniel

Duggal, Nisha

Dulgheroff, A.C.

Dumler, J Stephen

Dunachie, Susan

Dunn, JJ

Duparc, Stephan

Durand, Benoît

Durbin, Anna

Dusheiko, Geoffrey

Duthie, Malcolm

Eastwood, Gillian

Ebel, Gregory

Eberhard, Mark

Eckels, Kenneth

Eckmann, Lars

Emile, Charles

Emina, Jacques

Engman, David

Enwonwu, Cyril

Eppinger, Mark

Ercumen, Ayse

Eremeeva, Marina

Escalante, Ananias

Escolà Casas, Mònica

Estrada-Garcia, Teresa

Fairhurst, Rick

Falcone, Franco

Fan, CK

Faust, Christina

Ferrinho, Paulo

Fidock, David

Fierer, Joshua

Fischer, Peter

Fleischer, Bernhard

Fluegge, Kyle

Fonseca, Ana Gloria

Fooks, Anthony

Forrester, Naomi

Forshey, Brett

Fournier, Pierre

Foy, Brian

Franklin, Richard

Freedman, David

Freeman, Matthew

Frías Lasserre, Daniel

Fritz, Curtis

Frydman, Galit

Fujiwara, Ricardo

Fung, Isaac Chun-Hai

Gabriël, Sarah

Galactionova, Katya

Galan-Puchades, Maria Teresa

Galaviz Silva, Lucio

Galloway, Renee

Galvao, M.A.M

Gárate, Teresa

Garcia, Melissa

Garcia, Hector

García-Basteiro, Alberto L.

Garg, Ravindra Kumar

Garges, Eric

Gass, Katie

Gatti, Fabiane

Geoghegan, Patricia

George, Christine

Gérardin, Patrick

Ghany, Marc

Gibbons, Robert

Gil-Santana, Helcio

Gilliss, Catherine

Goh, Pik Pin

Gollob, Kenneth

Gomes, Suzete

Gonzalez, Lucia

González-Barrio, David

Good, Michael

Gorchakov, Rodion

Gordon, Aubree

Gosling, Roly

Goswami, Rama

Gottdenker, Nicole

Gradoni, Luigi

Graeff-Teixeira, Carlos

Graham, Elinor

Grant, Alison

Graves, Stephen

Graves, Patricia

Gray, Elizabeth

Green, Sharone

Green, Michael

Greenhouse, Bryan

Gregory, Christopher

Grobusch, Martin

Gromowski, Gregory

Grubaugh, Nathan

Grüner, Beate

Guerra, Marta

Guerrant, Richard

Guerrier, Gilles

Gurtler, Ricardo

Gutman, Julie

Gyawali, Pradip

Hachiya, Masahiko

Haddow, Andrew

Halliday, Jo

Halsey, Eric

Halstead, Scott

Hamano, Shinjiro

Hamer, Davidson

Hammitt, Laura

Hanington, Patrick

Hanrahan, Colleen

Hansen, Lawrence

Happi, Christian

Haque, Rashidul

Harris, Jason

Harrison, Mark

Hashemi, Mohammad

Hasker, Epco

Hassan, Habsah

Hearst, Norman

Heitzinger, Kristen

Heller, Tom

Henderson, Alden

Heo, Sang Taek

Hermans, Sabine

Herrington, James

Heyworth, Martin

Hien, Tran Tinh

Hill, Philip

Hobson-Peters, Jody

Hoffmann, Vivian

Hoft, Daniel

Holmen, Sigve

Hombach, Joachim

Homma, Akira

Hong, Sung-Tae

Hopkins, Adrian

Hoque, Bilqis

Horstick, Olaf

Hospenthal, Duane

Hossain, Mohammad

Hosseinipour, Mina

Hoyt, Alice

Hu, Wenbiao

Huang, Yan-Jang

Hubalek, Zdenek

Huddart, Sophie

Hugo, Leon

Hulland, Kristyna

Hung, Chien-Chin

Hungerford, Laura

Hunsperger, Elizabeth

Ifeanyi, Casmir

Inglis, Timothy

Irawati Ie, Susan

Isbister, Geoffrey

Ismail, Ahmad

Iwasaki, Hiromichi

Izurieta, Ricardo

Jackson, Alan

Jacobson, Raymond

Jaenisch, Thomas

Jagannathan, Prasanna

Jahrling, Peter

Jee, Youngmee

Jelinek, Tomas

Jennings, Zoe

Jeon, Hyeong-Kyu

Jia, Na

Jiang, Xiaolin

Jimenez-Coello, Matilde

Jit, Mark

Johansen, Kari

Johansson, Michael

John, Chandy

Johnson, Eric

Johnson, Reed

Jønsson, Rie

Julian, Tim

Juliano, Jonathan

Junglen, Sandra

Kabeya, Hidenori

Kachani, Malika

Kading, Rebekah

Kaestli, Mirjam

Kahawita, Indira

Kalayanarooj, Siripen

Kalimbira, Alexander A.

Kanda, Tatsuo

Kandi, Venkataramana

Kang, Gagandeep

Kansiime, Catherine

Karahan, Zeynep Ceren

Kargar, Faranak

Karmakar, Sumallya

Kathryn, Dupnik

Katrak, Shereen

Kelly, Daryl

Kelly, Paul

Kesselheim, Aaron

Kester, Kent

Khalil, Ibrahim

Khatchikian, Camilo

Kim, Yoon-Won

Kimanya, Martin

King, Charles

King, Christopher

Kirchhoff, Louis

Kirkpatrick, Beth

Klausner, Jeffrey

Klein, William

Klinger, Frederick

Klotz, Steve

Knust, Barbara

Kochar, Dhanpat

Kochel, Tadeusz

Kocken, Clemens

Koehler, Jane

Kohler, Pamela

Komar, Nicholas

Kondrashin, Anatoly

Konishi, Shoko

Koski, Kristine

Kramer, Laura

Krause, Peter

Kremer, Michael

Kropf, Pascale

Kruger, Detlev H.

Kuhn, Walter

Kumar, Pradeep

Kutsuna, Satoshi

L’Azou, Maïna

LaBeaud, Angelle Desiree

Labruna, Marcelo

Lacerda, Marcus

Laeyendecker, Oliver

Lai, Shengjie

Lam, Tommy

Lambert, Saba

Lambert, Amy

Lammie, Patrick

Lanata, Claudio

Lantagne, Daniele

Lanteri, Marion

Lantos, Paul

Larkins, Sarah

Larocque, Regina

Larsen, David

Larson, Heidi

Laskay, Tamas

Lau, Colleen

Laucella, Susana

Laurenti, Marcia

Le, Thuy

Leder, Kain

Lee, Chang-Seop

Lee, Won-Ja

Lee, Gwenyth

Lee, Keun Hwa

Lees, Rosemary

Lescano, Andres

Leski, Tomasz

Lesorogol, Carolyn

Levett, Paul

Levin, Michael

Levy, Barry

Lewnard, Joe

Ley, Benedikt

Li, Hao

Liang, Lili

Liautaud, Bernard

Libman, Michael

Libraty, Daniel

Lier, Tore

Lietman, Thomas

Lilje, Jonathan

Linder, Ewert

Lipsitch, Marc

Liu, Lecai

Lo, Shyh-Ching

Loker, Eric

Long, Kanya

Lopez, Velma

López, Manuel

Lopez-Verges, Sandra

Lover, Andrew

LoVerde, Philip

Lulitanond, Aroonlug

Luty, Adrian

Luvira, Viravarn

Lynch, Matthew

Mabey, David

Macaluso, Kevin

MacFarlane-Berry, Laura

Machado, Paulo

Mackenzie, Charles

Macpherson, Calum

Maes, Louis

Maes, Piet

Maggi, Ricardo

Magnaval, Jean-Francois

Mahajan, Sanjay

Mahapatra, Tanmay

Maleewong, Wanchai

Manary, Mark

Mansour, Adel

Marco, Jorge

Mariconti, Mara

Maritato, Maria

Marks, Michael

Martina, Byron

Mary, Charles

Matlashewski, Greg

Matsumura, Yusufumi

Mattioli, Mia Catharine

Mauricio, Isabel

Maurin, Max

McBain-Rigg, Kristin

McCarthy, James

McDonald, Emily

McDowell, Mary Ann

Mduluza, Takafira

Mehta, Sanjay

Mendis, Kamini

Menezes, Cristiane

Mesfine, Metadel

Meshnick, Steve

Michelet, Lorraine

Michelow, Ian

Minnick, Michael

Mintz, Eric

Miura, Kazutoyo

Miura, Tanya

Mlisana, Koleka

Modabber, Farrokh

Mokili, John

Molina, Israel

Molyneux, Elizabeth

Monath, Thomas

Mondal, Dinesh

Monecke, Stefan

Monteiro, Lorena

Monteiro, Fernando

Montgomery, Margaret (Maggie)

Montgomery, Susan

Moore, Catrin

Moore, Thomas

Moore, Sean

Moormann, Ann

Moraes, Milton

Morejón, Karen Mirna

Moro, Pedro

Morrison, Amy

Morse, Andrew

Mosqueda, Joel

Moss, William

Mota, Maria

Moutailler, Sara

Moyo, Sabrina

Mpimbaza, Arthur

Muehlenbachs, Atis

Mueller, Judith

Mueller, Ivo

Müller, Ingrid

Murphy, Edward

Murray, Henry

Murray, Kristy

Murthy, Rama

Mutebi, John-Paul

Nacher, Mathieu

Nadelman, Robert

Nagel, Robyn

Nakano, Tatsunori

Nakhla, Isabelle

Nardin, Elizabeth

Nash, Theodore

Nathan, Andre

Nelson, Kara

Nelson, Kenrad

Nelson, Julie

Neumayr, Andreas

Nevin, Remington

Newton, Paul

Ng, Lee Ching

Ngoma, Mombo

Ngure, Francis

Nicholson, William

Njabo, Kevin

Njenga, Sammy

Nogueira, Maurício

Noh, Susan

Noor, Abdisalan

Noordeen, Faseeha

Noordin, Rahmah

Null, Clair

Nunes, Marcio

Nutman, Thomas

Nylen, Susanne

O’Connell, Elise

O’Leary, Daniel

Obaro, Stephen

Oberhelman, Richard

Odermatt, Peter

Ogden, Nicholas

Ogden, Jessica

Ohta, Nobuo

Okumu, Fredros

Oliveira, Jacqueline

Oliveira, Jader

Olson, Ken

Ongerth, Jerry

Ortiz, Ernesto

Osier, Faith

Ostera, Graciela

Ott, Joerdis

Ottesen, Eric

Padgett, Kerry

Palmieri, James

Pan, Bo

Panosian Dunavan, Claire

Panzera, Francisco

Paris, Daniel

Pasaribu, Ayodhia

Patouillard, Edith

Pavia, Norma

Pearson, Richard

Pedroso, Enio

Peprah, Dorothy

Peredo, Rubén

Pereira, Luis

Perkins, Peter

Perkins, Alex

Peterson, Stefan

Piarroux, Renaud

Pinlaor, Somchai

Pinto, Mara

Pitisuttithum, Punnee

Polacheck, Itzhack

Pollett, Simon

Porter, Chad

Posey, Drew

Postels, Douglas

Pourette, Dolorès

Powers, Ann

Prakash, John

Premaratna, Ranjan

Prendergast, Andrew

Price, Ric

Price, Erin

Pritt, Bobbi

Queiroz-Telles, Flavio

Querido, Jailson

Quinnell, Rupert

Quispe, Antonio

Rabe, Ingrid

Ram, Pavani

Ramachandran, CP

Ramesh, V

Ranford-Cartwright, Lisa

Rao, Malla

Rao, Ramakrishna

Rastegar, Asghar

Rathod, Pradipsinh

Ravinetto, Raffaella

Ray, Sayantan

Read, Jennifer

Reed, Sharon

Reed, Steven

Regis, David

Rehman, Khalid

Reich, Nicholas

Reiner, Jr, Robert

Reisen, William K.

Reithinger, Richard

Reluga, Timothy

Requena, Pilar

Reves, Randall

Reyburn, Rita

Rhatigan, Joseph

Ribeiro, Aline

Richards, Frank

Richie, Thomas

Riddle, Mark

Riedel, David

Ringwald, Pascal

Ritmeijer, Koert

Rivera, Windell

Riviello, Elisabeth

Robertson, Gemma

Rocha, Fabiana

Rocha, Abraham

Rockabrand, David

Rockers, Peter

Rodriguez, Nilda

Rodriguez-Morales, Alfonso

Roess, Amira

Rogers-Brown, Jessica

Rogerson, Stephen

Rollin, Pierre

Romero, Gustavo

Root, Jeff

Roselino, Ana Maria

Rosenthal, Philip

Rosenthal, Joshua

Rothman, Alan

Rowland, Mark

Rowland-Jones, Sarah

Roxby, Alison

Roy, Sharon

Rudd, David

Rugemalila, Joas

Rugwizangoga, Belson

Rupprecht, Charles

Russell, Bruce

Ryan, Edward

Ryan, Edward, T.

Sack, David

Sacks, David

Sadhukhan, Provash

Sakru, Nermin

Salgado, Perla

Sanchez, Ana

Sanders, John

Santana, Alfredo

Santos, Ana Sofia

Santos, Victor

Saravia, Nancy

Sarfaty, Mona

Sarkar, Rajiv

Sarovich, Derek

Satoskar, Abhay

sauderson, Paul

Saunders, David

Savage, Harry

Schallig, Henk

Schell, Ellen

Schieffelin, John

Schmidt, Wolf-Peter

Schoepp, Randal

Schoeps, Anja

Schulze, Kerry

Schurz, Haiko

Schwab, Kellog

Schwan, Tom

Schwartz, Eli

Schwartz, Robert A.

Scollard, David

Scorpio, Diana

Scott, Alan

Seal, Andrew

Seas, Carlos

Secor, William

Seguro, Antonio

Sejvar, James

Seligman, Stephen

Senneville, Eric

Sereno, Denis

Serre, David

Severson, David

Sexton, Daniel

Shah, Maunank

Shakoor, Sadia

Shamikh, Yara

Shanks, Dennis

Sharma, Surya

Sharma, Vishal

Sharp, Tyler

Sharp, Tyler

Shelly, Mark

Shepard, Donald

Shevy, Laura

Shiff, Clive

Shim, Eunha

Shlim, David

Shu, Pei-Yun

Shwiff, Stephanie

Sibley, Carol

Sikazwe, Izukanji

Silaghi, Cornelia

Silberberg, Donald

Silpapojakul, Khachornsakdi

Silverman, Andrea

Simon, Fabrice

Simon, Gary

Singh, Om

Singh, SP

Sinha, Prabhat

Siqueira, Andre

Siriyasatien, Padet

Siyu, Sun

Slutsker, Laurence

Smith, Catherine

Smith, Robert

Smith, David

Smits, Henk

Smout, Michael

Song, Hummy

Sorvillo, Frank

Sotgiu, Giovanni

Sotillo, Javier

Sousa, Anastácio

Southern, Paul

Spears, Dean

Spencer, John

Ssengooba, Willy

Staedke, Sarah

Stamm, Lola

Standley, Claire

Stauber, Christine

Steinglass, Robert

Steinmann, Peter

Steinmetz, Ivo

Stenglein, Mark

Stensvold, Christen

Stevenson, Brian

Stewart, Christine

Stiles, Jonathan

Stojkovic, Marija

Stoltzfus, Rebecca

Stone, Geren

Strand, Tor

Strle, Franc

Stuart, Ken

Stuen, Snorre

Su, Yvonne

Sutherland, Colin

Suwantarat, Nuntra

Swetnam, Daniele

Sy, Francisco

Sydenham, Thomas

Tabachnick, Walter

Tai, Dar-In

Talaat, Kawsar

Talhari, Sinesio

Tan, Kevin S.

Tannich, Egbert

Tanowitz, Herbert

Tapia, Milagritos

Tarleton, Rick

Tarr, Phillip

Taylor, Diane

Taylor, Terrie

Taylor, Steve

Taylor, Walter

Taylor, Louise

Telford, Sam

Tesfahun, Esubalew

Tetteh, Kevin

Thaenkham, Urusa

Thaipadungpanit, Janjira

Thanassi, Wendy

Thiberville, SD

Thiele, Elizabeth

Thomas, Evan

Thompson, Amanda

Thriemer, Kamala Ley

Tielsch, James

Tilley, Drake

Tinoco-Gracia, Luis

Tisch, Daniel

Toledo, Rafael

Tong, Steven

Torgerson, Paul

Traub, Rebecca

Travi, Bruno

Troyo, Adriana

Tsakris, Athanassios

Tucker, Compton

Turner, Richard

Tyagi, Brij

Ubeira, Florencio

Udhayakumar, Venkatachalam

Unger, Holger

Unnasch, Thomas

Valecha, Neena

Valerio, Lluis

Valero, M. Adela

Van Borm, Steven

van den Hurk, Andrew

van der Hoek, Wim

van Griensven, Johan

Van Kerkhove, Maria

Van Panhuis, Willem

Varela-Stokes, Andrea

Vasilakis, Nikolaos

Vaz da Silva, Susana

Vazquez, Gonzalo

Venegas, Juan

Venegas, Aponte Erika

Vennervald, Birgitte

Vilela de Azeredo Oliveira, Maria Tercilia

Villar, Luis

Vincent, Veronique

Viney, Mark

Viola, George

Visser, Benjamin

Viswanathan, Stalin

Volkman, Sarah

von Groll, Andrea

von Seidlein, Lorenz

Vu, David

Waggoner, Jesse

Walker, David

Walker, Ned

Walsh, Douglas

Walsh, Sinead

Walson, Judd

Walter, Katharine

Wang, Chin-Chou

Wang, Alice

Warburg, Alon

Warkentien, Tyler

Warrell, David

Waters, Norman

Watts, Douglas

Weaver, Scott

Weidmann, Manfred

Weil, Gary

Weina, Peter

Weisenberg, Scott

Werneck, Guilherme

Wertheim, Heiman

Wesche, David

Wheeler, Sarah

White, A. Clinton

Whitehead, Stephen

Whittaker, Maxine

Wichaidit, Wit

Wickremasinghe, Renu

Wierzba, Thomas

Wijegunawardana, Asha

Wilder-Smith, Annelies

Wilkins, Patricia

Williams, David

Willoughby, Rodney

Wilson, Mary

Winglee, Kathryn

Wongsrichanalai, Chansuda

Wood, Heidi

Woods, Marion

Wormser, Gary

Wright, James

Wunder, Elsio

Wylie, Kristine

Wylie, Blair

Xu, Bianli

Yakubu, Ahmadu

Yamasaki, Hiroshi

Yamasaki, Hiroshi

Yanow, Stephanie

Yansouni, Cedric

Yeboah-Antwi, Kojo

Yen, Catherine

Yoon, In-Kyu

Yoshida, Tadashi

Young, JoAnne

Young, Edward

Yu, Xue-Jie

Zaman, Muhammad

Zaman, Sayed

Zambriski, Jennifer

Zavala-Castro, Jorge

Zeller, Herve

Zhang, Xingyu

Zhu, Guan

Zielinski-Gutierrez, Emily

Zompi, Simona

Zunt, Joseph

